# PET OWNERSHIP HISTORY AND SUCCESSFUL AGING OUTCOMES IN COMMUNITY-LIVING OLDER ADULTS

**DOI:** 10.1093/geroni/igz038.206

**Published:** 2019-11-08

**Authors:** Erika Friedmann, Nancy R Gee, Eleanor M Simonsick, Stephanie Studenski, Erik Barr, Barbara Resnick, Melissa H Kitner-Triolo, Alisha Hackney

**Affiliations:** 1 University of Maryland School of Nursing, Baltimore, Maryland, United States; 2 SUNY Fredonia, Fredonia, United States; 3 Longitudinal Studies Section, Intramural Research Program, National Institute on Aging, Baltimore, Maryland, United States; 4 NIA, Longitudinal Studies Section, Baltimore, Maryland, United States; 5 NIA, BRAIN AGING & BEHAVIOR SECTION, Baltimore, Maryland, United States

## Abstract

Diminishing cognitive and physical function, worsening psychological symptoms and increased mortality risk and morbidity typically accompany aging. Health needs of the aging population will continue to increase as the proportion of the population aged 50 years and older increases. Pet ownership (PO) has been linked to better health outcomes in older adults, particularly those with chronic health conditions. However, much of the evidence is weak with little known about the contribution of PO to successful aging in community living older adults. This study examines associations between PO and cognitive performance, physical functioning, and psychological status in community living older adults. Participants in the Baltimore Longitudinal Study of Aging (>50 years old, N=378) completed physical functioning, cognitive and psychological testing, and a ten-year PO history. Most participants (89%) had owned pets at some point and 24% currently have pets. Fourteen percent have 1-4 dogs, 12% have 1-4 cats, and 3% have others. PO was lower with older age (p<.001). In regression analysis controlling for age decade, pet ownership within the past 10 years (PO10) independently predicted cognitive function [total verbal learning (p=.04), and short (p=.015) and long (p=.031) delay free recall, ] but not physical function or psychological status. PO was lower in older age groups as was cognitive, physical functional, and psychological status, while PO within the past 10 years was associated with better verbal learning and memory independent of age. Longitudinal analysis is required to disentangle the sequential associations between PO and change in health status over time..

